#  Hospital and health administrator level barriers and priorities for National Neonatal Hearing Screening in Pakistan: A thematic analysis

**DOI:** 10.12669/pjms.36.5.1965

**Published:** 2020

**Authors:** Nazia Mumtaz, Ghulam Saqulain

**Affiliations:** 1 Dr. Nazia Mumtaz, PhD (Rehabilitation Sciences). Head of Department of Speech Language Pathology, Faculty of Rehabilitation & Allied Health Sciences, Riphah International University, Lahore, Pakistan; 2 Dr. Ghulam Saqulain, FCPS (Otorhinolaryngology). Head of Department, Department of Otorhinolaryngology, Capital Hospital, Islamabad, Pakistan

**Keywords:** Barriers, Disability, Hearing Impairment, Neonatal hearing screening, Public sector hospital

## Abstract

**Objectives:**

To investigate the Barriers and Priorities accorded by hospital and health administrators to neo-natal hearing screening.

**Methods:**

This qualitative exploratory descriptive study employing purposive sampling technique was conducted in Islamabad, over a period of 18 months from 1st August 2015 to 31st January 2017. Sample included the stakeholders i.e., Heads of public sector hospitals of Islamabad including Pakistan Institute of Medical Sciences, Capital Hospital, and Federal Government Services Hospital, Islamabad. Study included in depth interviews using a self-structured interview guide and audio recording. Recorded data was transcribed followed by thematic analyses which was manually drawn and verified.

**Results:**

The Outcomes from thematic analysis were drawn as Planning, Essential requirements for NNHS, High risk screening, education, Existence of skilled maternal & newborn health workers. Hearing screening equipment//facility instrumentation, Logistic support, Health ministry support and Financial cover are also significant outcomes. Lack of awareness in the public and professionals regarding the importance of early identification of HI, poor health infrastructure, burden on tertiary care and lack of referral top the list of barriers to initiation of NNHS program at hospital administrative level.

**Conclusion:**

The Barriers to NNHS identified at Hospital and Health care administrator level include lack of awareness, poor health infrastructure, burden on tertiary care and lack of referrals. Inherent barriers to NNHS cover the spectrum of lack of liaison/ linkages between obstetrics and other departments, deliveries at homes especially in rural areas, poor follow-up, scarcity of technical and adequately trained manpower. Intangible barriers to NNHS comprise lack of health care information system, attitudinal barriers, inadequate fiscal resources, and lack of integrated approach at intra departmental levels.

## INTRODUCTION

Neonatal Hearing Screening is vital to address hearing disability worldwide. There is a dearth of such programs in the middle and low income countries.^1^ National neonatal hearing screening (NNHS) programs are government level initiative to detect deafness interchangeably classified as hearing impairment from birth to one month of age. Such programs ensure intervention by six months of age, thus ensuring a child’s age appropriate speech language development, education and better quality of life.^2^

Pakistan has a huge population^3^ and with no neonatal hearing screening program is faced with delayed identification of hearing loss (HL).^4^ The barriers to NNHS in Pakistan extend at one end of the spectrum to the public sector hospital (PSH) and administrator’s level and at the other end at the policy maker’s level.

Deficiencies as regards comprehensive immunization programs, medical care, fiscal resources and etc are affecting the prevalence of HL in developing countries.^5^,^6^ In Pakistan lack of awareness among public and health care professionals, poor health infrastructure, burdened tertiary care, lack of referral and absence of liaison between obstetricians and other professionals persist. Home deliveries in rural areas, scarcity of technical manpower, lack of fiscal resource and attitudinal problems both at hospital administration and professional level are some of the barriers to NNHS. Compared to its population^3^, Pakistan’s health care faces structural, organizational and administrative checks on quality including control setups. Pakistan spends a total of 2.4% of GDP on healthcare as against an internationally recognized 4-5 % required to provide basic healthcare.^7^

With delayed identification of HL, cultural constraints, inadequate fiscal resource and trained healthcare manpower, Pakistan faces barriers in initiating NNHS. Hence this study was carried out to explore the barriers and priorities accorded by hospital and health administrators to neo-natal hearing screening. This study is very important due to dearth of literature on the subject. It is hoped that this study might appeal to those who matter and act as instigator to launch NNHS.

## METHODS

This is a qualitative exploratory descriptive study which employed purposive sampling for data collection. After approval of research by Institutional Research Committee (IIRS/Whom Concern. Letter/Student/2015/10/27/1513, Dated: 27th October 2015) of Isra University, study was carried out in the three public tertiary care hospitals of Islamabad including Pakistan Institute of Medical Sciences, Capital Hospital and Federal Government Services Hospital, Islamabad over a period of one and a half year from 1st August 2015 to 31st January 2017. The study sample included the stake holders i.e., the three administrative executive heads concerned with Hospital and Health care administration, who are heading the PSHs and with whom interviews were conducted with particular emphasis on NNHS. Due to the qualitative nature of enquiry, sample size was estimated to facilitate data saturation.

In depth interviews were the backbone of the research, which were conducted in English with pre-developed tested interview guide. The interview guide was tested on a medical doctor and a policy planner which helped access how discussion was led. Also the probes of each domain were built into the interview guide’s relevant area which steered the discussion into the domain areas. The questions were easily comprehensible with flow rearranged in a sequence. The researcher just provided enough direction to the respondents in order to address the element of digression, thus helping in exploration of the barriers, and allowed them to express their views uninhibited and at length.

The procedure followed for data collection started with a well-timed appointment and informed consent, followed by a detailed interview. This was digitally recorded and was initiated by putting forward queries regarding concepts of barriers. It helped in extraction of themes which were detailed and exhaustive. Participants were allowed to respond uninhibitedly, however, probes were resorted to where in depth information was necessary. Participants were allowed ample response time varying from half an hour to three hours.

Following data collection, content analysis was performed. The audio-recordings were transcribed and thematic analyses drawn manually. This was verified using two different coders including healthcare researcher and a planner. It started from areas of exploration of barriers to NNHS at policy level and broad codes were obtained, in which views & experiences were organized. The process of initial coding helped in identification of pattern and emergence of the initial themes. The coders independently identified themes and sub-themes within the transcripts. Differences noted in coding were discussed thoroughly and were solved by refining the definitions, creating new and collapsing low level codes. New themes that emerged from the collected data not fitting into agreed codes were assigned new codes. This followed fine-tuning so that results lead to the formation of consistent and rational arrangement.

## RESULTS

The current study revealed an array of rich information on the different barriers hindering NNHS in Pakistan. The thematic data analysis revealed as shown in Table-I, the main themes and their linkages to the characteristics that emerged during interviews followed by the barriers to NNHS that emerged following in depth interviews with hospital administrators (Table-II).

**Table-I:** Themes and Emerging Characteristics: Thematic Analysis.

S.No.	Outcome Themes	Characteristics
1	Planning	Administration of public health policy on sustainable pattern in a developed and mature hospital based NHS program. Provision of guidelines maturity of health care system, issuance of hospital initiated administrative orders. The uniform SOP’s , creation of digital data and record to gauge success of NNHS.
2	Essential requirements for NHS	In developing countries like Pakistan, presence of skilled maternal and newborn health workers, community-based screening to be linked to immunization clinics, Targeted hearing screening to be adopted, and liaison with UNHS organizations to be established.
3	High risk screening	Children missed using only high risk based screening, hearing screening equipment facility in every hospital along with maternity unit -an economically viable proposition, Centralized screening model.
4	Medical support	Primary prevention is rare. Acceptance of hearing impairment in medical community, knowledge and vision of primary physicians, Timely and appropriate diagnosis and treatment, Cooperation of primary and specialty health care providers.
5	Education	Create cell for advocacy and community based public awareness, Education of professionals including technicians and technical support staff, Education of the public including parents of newborn approaching or interacting with hospital.
6	Existence of skilled maternal & newborn health workers	Availability of skilled staff in tertiary care hospitals, availability in rural areas, availability of guidelines of neonatal hearing screening at all health care levels.
7	Hearing screening equipment facility/Instrumentation	Economic viability in developing countries like Pakistan, physiological tests of auditory function, behavior-based questionnaires to identify infants with HI in all populations
8	Logistic support	Training testing and follow-up by personnel, providing for screening laboratory operations (equipment, supplies and maintenance), maintaining appropriate records.
9	Health ministry support	Rectification of flaws in health care system by Ministry of Health Regulation and Coordination, present legislation & advocacy to support NNHS.
10	Financial cover	Economic burden, GDP for health screening as a whole, reliance of donor agencies.

**Table-II:** Barriers to NNHS at Hospital & Healthcare Administrator Level: Thematic Analysis.

S.No.	Barriers
1	Lack of awareness in both the public and the professionals regarding the importance of early identification of hearing impairment
2	Poor health infrastructure
3	Burden on tertiary care
4	Lack of referral
5	Lack of liaison/linkage between the obstetrics and other departments
6	Deliveries at homes especially in rural areas with the assistance of dais/other attendants
7	Poor follow-up bringing the initial efforts to halt
8	Scarcity of technical and adequately trained manpower
9	Lack of health care information system within hospital
10	Attitudinal barriers both at hospital administration and professional level
11	Inadequate fiscal resources
12	Lack of integrated approach at intra departmental levels

The outcomes drawn from the thematic analysis were Planning, Essential requirements for NNHS, High risk screening, Medical support, Education, Existence of skilled maternal & newborn health workers, Hearing screening equipment facility/Instrumentation, Logistic support, Health Ministry support and Financial cover.

## DISCUSSION

To explore the barriers to NNHS in Pakistan in Public Sector Hospitals (PSH), thematic analysis was conducted by interviewing administrators of federal government hospitals to ascertain the factors hindering the health care system. The findings of the current study was consistent with literature that newborns should be screened for HI (hearing impairment) so as to appreciate the most suitable manner to mainstream and rehabilitate neonates with any degree of hearing loss or hearing impairment traditionally classified as deafness by one month. Such newborns should be diagnosed before three months, in order to identify those infants whose Hearing loss (HL) is more than 40 decibels to bring down the age of identification of HL to 6 months for early intervention.^8^ Different studies have identified barriers like limited fiscal resource, manpower and support services shortage, lack of public awareness and uncertainty of healthcare provider’s commitment.^9^

In the present study, the hospital administrators stressed that NNHS in Pakistan could not be done until NNHS programs were in place in Public sector hospitals (PSH). In order to propagate lack of NNHS awareness has to be catered to. Similarly, in another study society’s insensitive attitude was reported as a barrier where media could effectively sensationalize issues like HI and NNHS.^10^ Such NNHS projects should be statistically endorsed case detection projections in controlled settings.^11^

The present study identified lack of referral system and contact between the primary specialty physician in PSH and the newborn. According to Hyde, systematic approach including timely identification through UNHS and follow up services are essential.^12^

In the current study lack of Health Care Information system based upon digital record postulates for audiological services and referrals to be combined with an effective follow up system to ensure a sustainable NNHS. Birth certificates can record a NNHS kit number for compliance monitoring. U.S Library of Medicine has simplified record keeping through standardized digitalization for ease of replication of NNHS systems.^11^

It was indicated in present study by hospital administrators that as adequately trained personnel were scarce, hence proper training of human resources should be carried out before launching NNHS. Winston and Ditty also noted shortage of rehabilitation professionals with expertise in pediatrics HL.^8^

The current study indicates that high prevalence of HI in the developing world was basically due to high birth rate and lack of sufficient documentation and diagnosis. Bulk of deliveries take place at homes in rural areas in hands of Daies with poor follow-up. Stevens G et al., in their study elaborated similar findings with high prevalence in middle and low income countries with delayed diagnosis^13^, though screening programs are cost effective ($ 10 to $ 50 per infant).^14^

This study highlights poor health infrastructure as a barrier indicating deficiencies in the community health care system which may be due to lack of primary prevention at district level hospitals. This may be due to advertence or otherwise by the hospital administrators attributable to huge daily influx patients at public sector tertiary care hospitals. Similarly, Nishtar S et al., in their study pointed out the insensitivity of these PSH.^15^

The present study also shows lack of a national integrated approach to NNHS with only province of Sindh having legislated on NHS in contrast to USA where 44 states have legislated. The low incidence of hospital births coupled with no complementary availability of skilled maternal and newborn health workers in the Pakistani health care system is distressing. Pakistan’s health care system comprises of 1167 hospitals, 5695 dispensaries, 5464 basic health units, 675 rural health centers, 733 mother & child health centers which remains inadequate for NHS.^16^ Similarly in case of Pacific Islands due to burden of infectious disease related childhood HI, acquired epidemiological data from an international health organization calls for strengthening the framework of primary health care and audiological services within the existing health care system.^17^

Our study supports the phenomena of age appropriate detection and early management of HI as in similar studies.^18^ Limited fiscal resource is also a Barrier.^5^ A study by Cunningham M et al.^19^ projected the notion children hailing from rural and low income background are at risk for HI especially because of paucity of funding as revealed in the present study.

In the current study, with the admittance of the hospital administrators, it has been established that no NNHS facility exists in PSH. Relevant health professionals are also not oriented towards detecting HL perhaps on account of an inadequate referral system in PSH. Similarly, an Australian study identified lack of any hospital based standard operating procedure despite available screening facilities with ENT departments with live births taking place daily in the same hospital. A child born in the hospital may return in a few months to a hospital and be diagnosed as HI having lost precious early intervention period.^20^

### Limitations of the study

The main limitation faced during the study was approaching and getting appointments from the participants. Recording of the interviews was also a hurdle for which lot of convincing was done.

## CONCLUSIONS

We conclude intrinsic barriers to NNHS in public sector hospitals in Pakistan loom in the shape of dire lack of human and financial resources. Barriers rise due to lack of awareness amongst health care professionals, planned execution of policies in a sustainable and standardized manner by federation and uniformity of approach. Infrastructure founded barriers to NNHS commonly are less or no availability of hearing screening equipment facility in every hospital. A desirable model of a centralized screening model should be housed con-jointly with pediatrics and gynecology units for detection of HI followed by referral, and its intervention. The barriers encompass factors including resource based impediments, scarcity of adequately trained manpower and lack of health care information system within hospitals. Aggravating factors such as attitudinal issues at administration level, inadequate fiscal resources and lack of integrated approach are intangible barriers to NNHS. Hence certain evidence based parameters and standards of cost effectiveness and detection criteria of NNHS programs need to be established prior to planning any NNHS program at public sector hospitals.

### Authors’ Contribution

**NM:** Conceptualization of work, designing of research, Data Analysis & Interpretation, Critical revision of article and responsible for accuracy & integrity of work.

**GS:** Writing of Manuscript, Methodology & Literature Review.
